# A Retrospective Comparison of Oncologic and Staging Outcomes Between Surgical Procedures–Video-Assisted Thoracoscopic Surgery Versus Thoracotomy in Pulmonary Adenocarcinoma

**DOI:** 10.3390/medicina62040702

**Published:** 2026-04-06

**Authors:** Bogdan Cosmin Tanase, Teodor Horvat, Alin Burlacu, Elena Chitoran, Vlad Rotaru, Traian Pătrașcu, Laurentiu Simion

**Affiliations:** 1 Medicine School, “Carol Davila” University of Medicine and Pharmacy, 050474 Bucharest, Romania; 2 Department of Thoracic Surgery, Bucharest Institute of Oncology “Prof. Dr. Alexandru Trestioreanu”, 022328 Bucharest, Romania; 3 General Surgery and Surgical Oncology Department I, Bucharest Institute of Oncology “Prof. Dr. Alexandru Trestioreanu”, 022328 Bucharest, Romania; 4 General Surgery Department, “Dr. I. Cantacuzino” Clinical Hospital, 030167 Bucharest, Romania

**Keywords:** pulmonary adenocarcinoma, video-assisted thoracic surgery, thoracotomy, lymph node dissection

## Abstract

*Introduction:* Lymph node status is a key prognostic factor of lung cancer. Although video-assisted thoracoscopic surgery (VATS) is widely used for early-stage disease, its consistency in achieving thorough lymph node dissection remains debated. While many studies show outcomes comparable to thoracotomy, others question its reliability for accurate staging in advanced cases. This study compared the oncologic efficacy of VATS and thoracotomy in pulmonary adenocarcinoma, focusing on lymph node dissection and postoperative outcomes. *Materials and Methods:* A retrospective analysis was conducted on 111 consecutive patients who underwent curative-intent resection for pulmonary adenocarcinoma between 2019 and 2023 at the “Prof. Dr. Alexandru Trestioreanu” Oncological Institute, 52 undergoing thoracotomy and 59 Video-Assisted Thoracoscopic Surgery (VATS). *Results:* Demographic and clinical characteristics were comparable between groups. Compared with thoracotomy, VATS was associated with a significantly higher number of harvested lymph nodes at stations 7 and 10. No significant differences between groups in the number of positive lymph nodes, postoperative morbidity, or 30-day mortality were observed. *Conclusions:* VATS appears to provide comparable lymph node retrieval and short-term outcomes to open surgery. These findings add valuable data from an underrepresented Eastern European population and support the broader adoption of minimally invasive techniques in lung cancer surgery.

## 1. Introduction

Lung cancer is one of the most frequently diagnosed malignancies worldwide and remains the leading cause of cancer-related mortality, accounting for approximately 1.8 million fatalities annually [1]. Lymph node status is one among the most important prognostic determinants in non-small cell lung cancer (NSCLC), directly influencing survival and therapeutic strategies. Accurate staging through systematic lymphadenectomy therefore remains essential for optimal treatment planning and prognostic stratification.

Traditionally, thoracotomy has been the standard approach for surgical resection and systematic lymphadenectomy in NSCLC; however, the last two decades have witnessed a paradigm shift toward minimally invasive techniques. Video-assisted thoracoscopic surgery (VATS) has emerged as a less invasive alternative to open surgery, offering advantages such as shorter hospital stays, reduced postoperative pain, lower complication rates, and faster recovery to baseline function [2]. Nevertheless, concerns persist regarding the oncological adequacy of VATS, particularly its ability to achieve a lymph node dissection comparable to that of open thoracotomy. While several studies have demonstrated equivalent oncologic outcomes in early-stage NSCLC, others have questioned its reliability for accurate staging and nodal clearance in more advanced disease.

The present study aims to compare the oncological efficacy of VATS and thoracotomy in patients with pulmonary adenocarcinoma, focusing specifically on lymph node yield, staging accuracy, and short-term postoperative outcomes. By evaluating these parameters, we seek to determine whether VATS can be considered an oncological equivalent alternative to thoracotomy for this common and aggressive malignancy.

## 2. Materials and Methods

### 2.1. Study Design

This study was designed as a retrospective, observational, comparative analysis of patients diagnosed with pulmonary adenocarcinoma who underwent surgical treatment at the “Prof. Dr. Alexandru Trestioreanu” Oncological Institute in Bucharest, Romania, between January 2019 and December 2023. The study protocol received approval from the Institutional Ethics Committee (approval number 211/19 December 2024), and all research was conducted in accordance with the principles of the Declaration of Helsinki.

Informed consent from individual patients was considered not necessary given the retrospective nature of our study. Even so, all patients admitted for treatment in our institution sign a general consent form permitting the anonymous usage of medical data and results for clinical research, educational purposes and for developing and publishing scientific publications.

The primary endpoints of this study were lymph node yield, staging accuracy, and short-term postoperative outcomes. Long-term oncologic outcomes, such as disease-free survival and overall survival, were not included due to variability in follow-up duration and incomplete data availability.

### 2.2. Study Population and Inclusion Criteria

In our study we included all consecutive patients who underwent curative-intent resection for pulmonary adenocarcinoma. Patients were considered eligible for inclusion if they had a histologically confirmed diagnosis of primary pulmonary adenocarcinoma and underwent anatomical lung resection with systematic lymph node dissection via either open thoracotomy or video-assisted thoracoscopic surgery (VATS).

Exclusion criteria included patients undergoing non-anatomical resections (i.e., wedge resections), regardless of lymphadenectomy status, as well as procedures without systematic lymph node dissection and cases with incomplete surgical or histopathological records.

The final study cohort was comprised of 111 consecutive patients, with pulmonary cancer stages IA–IIIB. All cases were discussed within a multidisciplinary tumor board prior to surgery.

Of those, 52 underwent open thoracotomy, and 59 underwent VATS. All procedures were performed by experienced thoracic surgeons within the same institution, following standard oncologic principles. The choice of surgical approach (VATS vs. thoracotomy) was not randomized and was based on tumor characteristics, patient comorbidities, technical considerations, and surgeon expertise. While not all surgeons performed VATS uniformly, all had substantial experience in thoracic oncologic surgery. All VATS procedures were performed using a multiportal approach.

Complex cases such as centrally located tumors, extensive hilar or mediastinal adhesions, or suspected vascular involvement were preferentially treated by thoracotomy, while VATS was considered for peripherally located tumors and patients with favorable anatomy.

### 2.3. Preoperative Evaluation

All patients underwent a standardized preoperative assessment, including clinical examination, pulmonary function tests (with forced expiratory volume in 1 s—FEV1) and echocardiography (for left ventricular ejection fraction). Preoperative staging included contrast-enhanced computed tomography (CT) of the chest and upper abdomen. Positron emission tomography–CT (PET-CT) was performed selectively electively based on clinical indication and resource availability. Brain imaging (CT or MRI) was performed selectively based on clinical suspicion or advanced disease stage. Cardiopulmonary risk was assessed using the American Society of Anesthesiologists (ASA) physical status classification and the Charlson Comorbidity Index (CCI).

ASA physical status score was used to assess preoperative functional status and anesthetic risk. Patients were classified preoperatively into ASA class I–V based on their systemic health and comorbid conditions: ASA II—Mild systemic disease (e.g., well-controlled hypertension or diabetes), and ASA III—Severe systemic disease limiting activity but not incapacitating (e.g., unstable angina, chronic obstructive pulmonary disease). No patients in ASA I, IV or V were included in this cohort.

CCI was used to quantify the burden of pre-existing comorbidities for each patient. The CCI assigns a weighted score (from 1 to 6) to a predefined set of comorbid conditions, including but not limited to myocardial infarction, congestive heart failure, diabetes, chronic pulmonary disease, renal impairment, and malignancy. The total CCI score is obtained by summing the weights of all present conditions. In this study, patients were stratified into two groups based on comorbidity burden: CCI < 4 (low-to-moderate risk) and CCI ≥ 4 (high-risk), as previously validated in surgical outcome research.

### 2.4. Surgical Procedures

Anatomical resections were performed according to tumor location and extent, including lobectomy and segmentectomy. Systematic lymphadenectomy was carried out in all cases, aiming to harvest lymph nodes from stations 2R/4R, 4L, 5, 6, 7, 9, and 10, in line with current guidelines for NSCLC. The surgical approach (open vs. VATS) was recorded, along with intraoperative variables such as operative time, estimated blood loss, and transfusion requirement.

### 2.5. Postoperative Evaluation

Postoperative complications were classified according to the Clavien-Dindo grading system. The length of intensive care unit (ICU) stay, total hospitalization duration, and 30-day mortality were recorded for each patient. Pathological data were extracted from final histology reports, including tumor size, pathological staging (TNM classification, 8th edition), number of retrieved lymph nodes per station, and number of metastatic nodes.

### 2.6. Statistical Analysis

Statistical analysis was performed using SPSS version 22.0 (IBM Corp., Armonk, NY, USA), and additional analyses were conducted using Python v3.12. Continuous variables were presented as means with standard deviations or medians with ranges, depending on data distribution. Categorical variables were expressed as frequencies and percentages.

The distribution of continuous variables was assessed using the Shapiro–Wilk test prior to selecting parametric or non-parametric statistical tests. Comparisons between the two groups (open vs. VATS) were conducted using the independent *t*-test or Mann–Whitney U test for continuous variables and the chi-square or Fisher’s exact test for categorical variables. A *p*-value < 0.05 was considered statistically significant.

A multivariable logistic regression analysis was performed to evaluate factors associated with the choice of surgical approach, including age, comorbidity burden (CCI), clinical stage, and neoadjuvant therapy.

## 3. Results

### 3.1. Baseline Patient Characteristics

A total of 111 consecutive patients with pulmonary adenocarcinoma underwent surgical resection during the study period. The mean age at the time of surgery was 61 years (range: 29–82 years). Of these, 52 patients were treated via open thoracotomy, while 59 underwent VATS.

Comparative analysis of demographic and clinical characteristics revealed no statistically significant differences between the two groups in terms of age, sex distribution, comorbidity burden (as assessed by the CCI), ASA physical status, exposure to toxic agents, or preoperative cardiac and pulmonary function. Tumor location and preoperative clinical stage appeared broadly comparable between groups in univariate analysis.

However, a significant difference was observed regarding the type of surgical resection performed. Segmentectomy was performed more frequently in the VATS group than in the open surgery group (13 patients vs. 2 patients, respectively; *p*-value = 0.005). This likely reflects a preference for minimally invasive approaches in patients with smaller, peripherally located tumors suitable for sub-lobar resection. A detailed summary of the demographic and clinical data is provided in Table 1.

Since the inclusion of patients who received neoadjuvant chemotherapy may introduce a potential source of bias, as these cases are often associated with more advanced disease, we performed a sensitivity analysis excluding these 10 patients. The main findings remained consistent, with no significant differences observed between groups in lymph node yield or postoperative outcomes.

Also, a multivariable logistic regression analysis was performed to explore factors associated with the choice of surgical approach. Clinical stage was independently associated with the use of VATS, with minimally invasive surgery being more frequently performed in earlier-stage disease (OR = 0.36, *p*-value = 0.02). No other variables, including age, comorbidity burden, or neoadjuvant therapy, were independently associated with surgical approach.

Although a multivariable analysis was performed, residual confounding related to unmeasured variables and inherent selection bias cannot be fully excluded.

### 3.2. Intraoperative and Pathological Findings

Intraoperative parameters, including the duration of the surgical procedure, estimated blood loss, and the need for perioperative blood transfusion, were recorded for all patients. The type of anatomical resection was also documented. A detailed comparison of these variables and histopathological findings between the open surgery and VATS groups is presented in Table 2.

As shown in Table 2, the median number of lymph nodes retrieved from stations 7 and 10 was significantly higher in patients undergoing VATS compared to those treated by thoracotomy with open surgery (*p*-value = 0.0001 and *p*-value = 0.0162, respectively). This suggests a potential advantage of the minimally invasive approach in terms of nodal yield, particularly in the subcarinal and hilar regions. However, there was no statistically significant differences between the two groups regarding the number of metastatic (invaded) lymph nodes identified at any station, indicating comparable oncological clearance.

The distribution of final pathological stages was similar between groups (*p*-value = 0.727).

**Figure 1:**
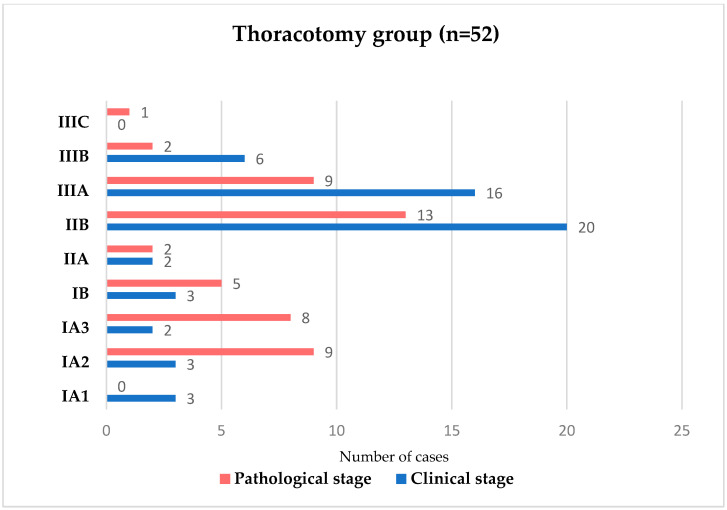
Comparison between clinical staging and pathological restaging distribution in patients undergoing thoracotomy.

**Figure 2:**
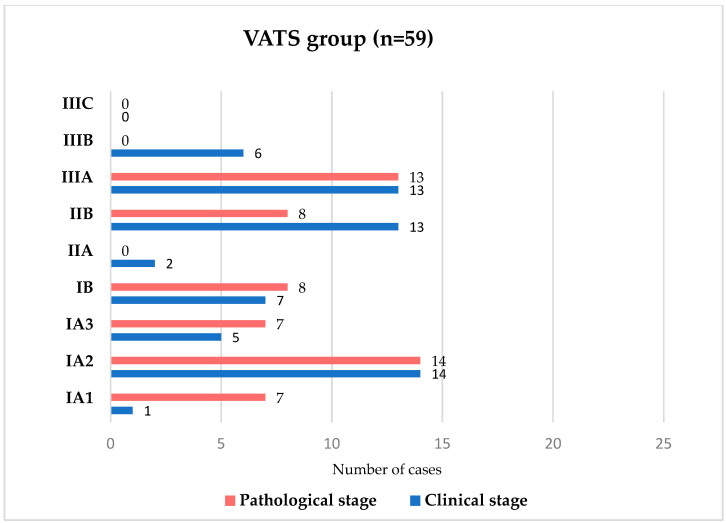
Comparison between clinical staging and pathological restaging distribution in patients undergoing VATS.

Restaging analysis from initial clinical stage to final pathological stage is illustrated in Figure 1 (open surgery) and Figure 2 (VATS).

Upstaging from clinical to pathological stage was observed more frequently in the VATS group, whereas under-staging was more common following thoracotomy. While this may suggest differences in nodal assessment, this finding should be interpreted with caution. Variations in baseline characteristics and surgical selection criteria between groups may have influenced these results. Therefore, nodal upstaging should not be considered a direct indicator of superior staging accuracy, but rather a potentially multifactorial finding that warrants further investigation.

### 3.3. Postoperative Outcomes

Postoperative recovery was uneventful in 65.8% of patients. The median ICU stay was 1 day, while the median duration of hospitalization was 9 days across the entire cohort. The overall postoperative morbidity rate was comparable between groups: 32.6% after open surgery and 35.6% after VATS (*p*-value = 0.714), indicating no significant difference in short-term complication rates. Importantly, there were no deaths within 30 days postoperatively in either group.

A detailed summary of postoperative outcomes, including complication grading according to the Clavien-Dindo classification, is presented in Table 3.

## 4. Discussion

VATS is among the procedures aimed at de-escalating the extent of surgery and limiting the overall impact on morbidity and patient comfort [3,4,5,6]. Lymph node status is a critical prognostic factor in pulmonary adenocarcinoma, directly influencing survival and adjuvant therapy. Accurate staging, however, remains challenging. CT has limited sensitivity, particularly for non-enlarged nodes, with reported diagnostic accuracy ranging from 45% to 79% [7,8]. More invasive procedures such as mediastinoscopy, endobronchial ultrasound, or fine-needle aspiration improve accuracy but remain operator-dependent and may yield significant false-negative results [9,10].

Minimally invasive surgery has revolutionized thoracic oncology, offering reduced pain, faster recovery, and oncological outcomes comparable to open surgery. Since the first report of VATS lymph node dissection in 1994 [11], multiple studies have confirmed its feasibility and perioperative advantages, including lower morbidity and better quality of life compared with thoracotomy [12,13]. A meta-analysis of 16 studies (44,673 patients) found similar nodal yields between VATS and thoracotomy, with slightly better disease-free and overall survival in the VATS cohort [14]. Complete mediastinal lymph node dissection remains crucial, as it correlates with improved long-term outcomes [15,16,17].

In our series, VATS retrieved significantly more nodes at stations 7 and 10 compared with thoracotomy, likely due to enhanced visualization and dissection accuracy. Although the number of metastatic nodes did not differ, this higher yield may reflect differences in surgical technique or exposure, although it should be interpreted with caution given the potential influence of selection bias.

The multivariable analysis confirmed that clinical stage influenced the choice of surgical approach, with VATS being preferentially used in earlier-stage disease. This finding supports the presence of selection bias inherent to the retrospective design and should be considered when interpreting comparative outcomes.

Importantly, the absence of long-term oncologic outcomes, such as disease-free and overall survival, limits the ability to draw definitive conclusions regarding oncological equivalence between the two surgical approaches. Therefore, the present findings should be interpreted primarily in the context of staging adequacy and short-term outcomes.

Complication rates and hospital stay duration were comparable between groups, supporting the feasibility of achieving comprehensive nodal dissection via VATS without compromising safety. The minimally invasive nature of VATS may also facilitate earlier recovery and timely initiation of adjuvant therapy when indicated. The slightly longer hospitalization in both groups compared to international data (12 days for open surgery and 13 days for VATS) reflects institutional and national practices, not inferior perioperative outcomes.

Upstaging following VATS may reflect improved detection of occult nodal disease; however, this finding should be interpreted with caution. Differences in baseline characteristics and surgical selection between groups may have contributed to this observation. Therefore, nodal upstaging cannot be considered a direct indicator of superior staging accuracy, but rather a potentially multifactorial finding. Previous reports similarly show VATS as feasible even after neoadjuvant chemotherapy for stage IIIA disease, without compromising margins or safety [18]. Nevertheless, concerns remain about adequacy of mediastinal dissection, particularly in superior stations (2R, 4R). A meta-analysis by Chen et al. reported slightly lower nodal harvests with VATS compared to thoracotomy, though the clinical impact is uncertain [19]. In our series, VATS yielded more nodes at stations 7 and 10, highlighting the role of surgeon experience and technique optimization.

Segmentectomy, more frequently performed in the VATS group in our series, reflects a global trend toward parenchyma-sparing resections in early-stage adenocarcinoma. Recent randomized trials, including JCOG0802/WJOG4607L and CALGB/Alliance 140503, have demonstrated non-inferior—and in some cases superior—overall survival for anatomical segmentectomy compared with lobectomy in selected stage IA NSCLC, provided that adequate margins and systematic lymph node dissection are achieved [20,21]. However, sub-lobar resections require meticulous nodal assessment to avoid under-staging, particularly when performed via minimally invasive approaches. Our findings of higher nodal yield with VATS suggest that adequate nodal assessment can be achieved in selected patients, although these results should be interpreted cautiously due to potential selection bias.

Large registry analyses further support these findings. A propensity-matched study from the STS database reported improved 5-year overall survival and reduced morbidity with VATS lobectomy compared to thoracotomy [22], particularly benefiting elderly and comorbid patients through fewer respiratory complications and faster mobilization [23]. Our comparable morbidity and zero 30-day mortality are consistent with these results [12,13,14,22,23].

The success of VATS depends heavily on surgeon experience and institutional expertise. High procedural volume correlates with superior lymph node retrieval, lower complication rates, and improved overall outcomes [24]. Therefore, structured training and quality control are essential for reproducible results across institutions.

Technically, VATS offers magnified visualization and angled optics enabling meticulous subcarinal and hilar lymph node dissection. This may explain our higher nodal yields at stations 7 and 10. Conversely, thoracotomy allows manual palpation and better access to mediastinal nodes (2R and 4R). Both approaches can achieve adequate oncological clearance when performed by experienced surgeons and appropriately selected for tumor location.

Accurate pathological staging has become increasingly important with the advent of perioperative immunotherapy and targeted treatments. The NADIM trial highlighted the value of postoperative nodal assessment for identifying candidates for adjuvant immunotherapy, especially in resectable stage IIIA disease [25]. In this context, VATS offers not only a less invasive option, but also a platform capable of delivering high-quality oncologic staging. Likewise, the ADAURA and IMpower010 trials demonstrated survival benefits of adjuvant Osimertinib and Atezolizumab, emphasizing the need for complete surgical staging to guide therapy [20,26]. Current expert consensus guidelines from the Society of Thoracic Surgeons recommend VATS as the preferred surgical approach for stage I NSCLC, citing equivalent oncological efficacy and lower perioperative morbidity compared to thoracotomy [27]. Nodal upstaging following VATS—defined as the unexpected identification of N1 or N2 disease in patients clinically staged as N0—has been increasingly recognized as a relevant prognostic factor, underscoring the importance of thorough lymph node dissection even in minimally invasive settings [28].

While robotic-assisted thoracic surgery (RATS) may overcome some technical limitations of VATS, multicenter data indicate equivalent nodal yields and outcomes when performed by experienced teams [29]. However, RATS is not yet available in our institution and remains unavailable in many Eastern Europe centers. Therefore, VATS continues to represent the standard minimally invasive approach and remains the main comparator to thoracotomy in daily practice. Presenting real-world data from such settings is therefore relevant, reflecting current clinical decision-making and resource availability.

### Study Limitations

The present study has several limitations. First, its retrospective design and the absence of randomization introduce a potential risk of selection bias, particularly regarding the allocation of surgical approach. Second, the relatively small sample size and limited number of events restricted the feasibility of performing robust multivariable or propensity score–adjusted analyses, as such models would be prone to overfitting and unreliable estimates. Also, although a sensitivity analysis excluding the patients with neoadjuvant therapy yielded similar results, the inclusion of such cases may still represent a potential source of bias. Third, long-term oncologic outcomes such as disease-free and overall survival were not consistently available due to variability in follow-up duration. As a result, the present study cannot provide conclusions regarding long-term survival or recurrence, and the findings should be interpreted in the context of short-term and staging-related outcomes. Therefore, the findings of this study should be interpreted as exploratory and hypothesis-generating within the context of these limitations.

## 5. Conclusions

VATS appears to be a safe and effective surgical approach for pulmonary adenocarcinoma, providing comparable lymph node retrieval and short-term postoperative outcomes to open thoracotomy in this cohort.

While these findings support the feasibility of minimally invasive techniques in routine clinical practice, the results should be interpreted with caution given the retrospective design and potential selection bias. Further studies incorporating long-term oncologic outcomes and adjusted analyses are required to confirm these observations.

In addition to its oncological adequacy, the minimally invasive nature of VATS contributes to reduced postoperative discomfort, shorter hospital stays, and faster return to baseline functional status. These advantages may also enable earlier initiation of adjuvant therapies when indicated, potentially improving long-term outcomes.

While VATS and thoracotomy have been compared in previous studies, our unicentric analysis focused on pulmonary adenocarcinoma in an Eastern European setting provides practical, real-world insights, thus adding value to the existing body of literature.

## Figures and Tables

**Table 1:** Demographic and clinical data of the patients diagnosed with pulmonary adenocarcinoma, sub-mitted to open versus video-assisted surgery.

Variable	Open Approach (*n* = 52 Cases)	VATS (*n* = 59 Cases)	*p*-Value
**Age (mean, years)**	60.7	61.7	0.307
**Sex**			0.894
Male	24	29	
Female	28	30	
**ASA score**			0.830
II	13	16	
III	39	43	
**CCI**			0.820
≤4	28	33	
>4	24	26	
**Exposure to toxic agents**			0.511
Yes	6	4	
No	46	55	
**FEV1 (mean)**	79	83	0.260
**Left ventricle ejection fraction (mean)**	60%	55%	0.540
**Tumor site**			0.723
LLL	11	6	
LUL	12	20	
RLL	12	9	
RML	4	4	
RUL	13	20	
**Clinical stage**			0.791
IA1	0	1	
IA2	3	14	
IA3	2	5	
IB	3	7	
IIA	2	0	
IIB	20	13	
IIIA	16	13	
IIIB	6	6	
**Type of resection**			**0.005**
Lobectomy	50	46	
Segmentectomy	2	13	
**History of NAT**			0.125
Yes	7	3	
No	45	56	

Abbreviations: CCI, Charlson Comorbidity Index; FEV1, forced expiratory volume in one second; ASA, intraoperative American Society of Anesthesiologists physical status classification; LLL, Left Lower Lobe; LUL, Left Upper Lobe; RLL, Right Lower Lobe; RML, Right Middle Lobe; RUL, Right Upper Lobe; VATS, video-assisted thoracoscopic surgery; NAT, neoadjuvant therapy. Bold text represents the subcategories.

**Table 2:** Intraoperative and histopathological data of the patients in the open approach and VATS groups.

Variable	Open Approach (*n* = 52 Cases)	VATS (*n* = 59 Cases)	*p*-Value
**Number of retrieved lymph nodes from stations (median and range)**			
2R + 4R	2 (0–9)	2 (0–14)	0.1134
4L	0 (0–2)	0 (0–2)	0.0893
5	0 (0–4)	0 (0–5)	0.1646
6	0 (0–3)	0 (0–3)	0.1521
7	0 (0–6)	4 (0–13)	**0.0001**
9	1 (0–9)	1 (0–2)	0.0867
10	4 (0–9)	4 (1–12)	**0.0162**
**Number of invaded lymph nodes (median and range)**			
2R + 4R	0 (0–3)	0 (0–2)	0.2067
4L	0 (0)	0 (0)	0.5000
5	0 (0)	0 (0–2)	0.0525
6	0 (0)	0 (0–1)	0.4644
7	0 (0–2)	0 (0–3)	0.2441
9	0 (0–1)	0 (0)	0.5001
10	0 (0–4)	0 (0–8)	0.3036
**Pathological stage**			0.7272
IA1	3	7	
IA2	9	14	
IA3	8	7	
IB	5	8	
IIA	2	2	
IIB	13	8	
IIIA	9	13	
IIIB	2	0	
IIIC	1	0	

Abbreviations: VATS, video-assisted thoracoscopic surgery. Bold text represents the subcategories.

**Table 3:** Evolution data of the patients diagnosed with pulmonary adenocarcinoma, submitted to open versus video-assisted surgery.

Variable	Open Approach (*n* = 52 Cases)	VATS (*n* = 59 Cases)	*p*-Value
**ICU stay (days, median)**	2.46	3.46	0.243
**Postoperative hospital stay (days, median)**	12.17	13.76	0.269
**Complications (Clavien-Dindo)**			0.714
Grade I	4	7	
Grade II	7	9	
Grade IIIA	2	3	
Grade IIIB	1	1	
Grade IVA	2	0	
Grade IVB	1	1	
**Postoperative complication rate**	32.6%	35.6%	0.840
**30 days postoperative mortality rate**	0	0	

Abbreviations: ICU, Intensive care unit; VATS, video-assisted thoracoscopic surgery. Bold text represents the subcategories.

## Data Availability

Data is available on reasonable request from A.B.

## Abbreviations

The following abbreviations are used in this manuscript:

VATS	Video-Assisted Thoracoscopic Surgery
NSCLC	Non-small cell lung cancer
FEV1	Forced expiratory volume in 1 s
CT	Computed tomography
PET-CT	Positron emission tomography
ASA	American Society of Anesthesiologists
CCI	Charlson Comorbidity Index
ICU	Intensive Care Unit
LLL	Left Lower Lobe
LUL	Left Upper Lobe
RLL	Right Lower Lobe
RML	Right Middle Lobe
RUL	Right Upper Lobe
NAT	Neoadjuvant therapy

## References

[1] Siegel R.L., Miller K.D., Fuchs H.E., Jemal A. (2021). Cancer Statistics, 2021. CA Cancer J. Clin..

[2] Nitadori J.I., Bograd A.J., Kadota K., Sima C.S., Rizk N.P., Morales E.A., Rusch V.W., Travis W.D., Adusumilli P.S. (2013). Impact of micropapillary histologic subtype in selecting limited resection vs lobectomy for lung adenocarcinoma of 2cm or smaller. J. Natl. Cancer Inst..

[3] Rotaru V., Chitoran E., Cirimbei C., Cirimbei S., Simion L. Preservation of Sensory Nerves During Axillary Lymphadenectomy. Proceedings of the 35th Balkan Medical Week.

[4] Cirimbei C., Rotaru V., Chitoran E., Cirimbei S. Laparoscopic Approach in Abdominal Oncologic Pathology. Proceedings of the 35th Balkan Medical Week.

[5] Chitoran E., Rotaru V., Mitroiu M.N., Durdu C.E., Bohiltea R.E., Ionescu S.O., Gelal A., Cirimbei C., Alecu M., Simion L. (2024). Navigating Fertility Preservation Options in Gynecological Cancers: A Comprehensive Review. Cancers.

[6] Cirimbei C., Rotaru V., Chitoran E., Pavaleanu O., Cirimbei S.E. (2017). Immediate and Long-term Results of Radiofrequency Ablation for Colorectal Liver Metastases. Anticancer Res..

[7] Steinert H.C., Hauser M., Allemann F., Engel H., Berthold T., Von Schulthess G.K., Weder W. (1997). Non-small cell lung cancer: Nodal staging with FDG PET versus CT with correlative lymph node mapping and sampling. Radiology.

[8] Prenzel K.L., Mo S.P., Sinning J.M., Baldus S.E., Brochhagen H.G., Schneider P.M., Ho A.H. (2003). Lymph node size and metastatic infiltration in non-small cell lung cancer. Chest.

[9] Gilart J.F., Gámez García P., Rodríguez De Castro F., Suárez P.R., Rodríguez N.S., Varela De Ugarte A. (2000). Extended cervical mediastinoscopy in the staging of bronchogenic carcinoma. Ann. Thorac. Surg..

[10] Annema J.T., Veseliç M., Versteegh M.I.M., Willems L.N.A., Rabe K.F. (2003). Mediastinal restaging: EUS-FNA offers a new perspective. Lung Cancer.

[11] McKenna R.J. (1994). Lobectomy by video-assisted thoracic surgery with mediastinal node sampling for lung cancer. J. Thorac. Cardiovasc. Surg..

[12] Onaitis M.W., Petersen R.P., Balderson S.S., Toloza E., Burfeind W.R., Harpole D.H., D’Amico T.A. (2006). Thoracoscopic Lobectomy Is a Safe and Versatile Procedure. Ann. Surg..

[13] Bendixen M., Jørgensen O.D., Kronborg C., Andersen C., Licht P.B. (2016). Postoperative pain and quality of life after lobectomy via video-assisted thoracoscopic surgery or anterolateral thoracotomy for early stage lung cancer: A randomised controlled trial. Lancet Oncol..

[14] Nath T.S., Mohamed N., Gill P.K., Khan S. (2022). A Comparative Analysis of Video-Assisted Thoracoscopic Surgery and Thoracotomy in Non-Small-Cell Lung Cancer in Terms of Their Oncological Efficacy in Resection: A Systematic Review. Cureus.

[15] Jeon H.W., Moon M.H., Kim K.S., Du Kim Y., Wang Y.P., Park H.J., Park J.K. (2014). Extent of Removal for Mediastinal Nodal Stations for Patients with Clinical Stage I Non-Small Cell Lung Cancer: Effect on Outcome. Thorac. Cardiovasc. Surg..

[16] David E.A., Cooke D.T., Chen Y., Nijar K., Canter R.J., Cress R.D. (2017). Does Lymph Node CountInfluence Survival in Surgically Resected Non-Small Cell Lung Cancer?. Ann. Thorac. Surg..

[17] Ou S.-H.I., Zell J.A. (2008). Prognostic Significance of the Number of Lymph Nodes Removed at Lobectomy in Stage IA Non-small Cell Lung Cancer. J. Thorac. Oncol..

[18] Yang H.X., Woo K.M., Sima C.S., Bains M.S., Adusumilli P.S., Huang J., Finley D.J., Rizk N.P., Rusch V.W., Jones D.R. (2017). Long-term Survival Based on the Surgical Approach to Lobectomy For Clinical Stage I Nonsmall Cell Lung Cancer. Ann. Surg..

[19] Chen F.F., Zhang D., Wang Y.L., Xiong B. (2013). Video-assisted thoracoscopic surgery lobectomy versus open lobectomy in patients with clinical stage I non-small cell lung cancer: A meta-analysis. Eur. J. Surg. Oncol..

[20] Altorki N.K., Wang X., Wigle D., Gu L., Darling G., Ashrafi A.S., Landrenau R., Miller D., Liberman M., Jones D.R. (2018). Perioperative mortality and morbidity after sublobar versus lobar resection for early-stage non-small-cell lung cancer: Post-hoc analysis of an international, randomised, phase 3 trial (CALGB/Alliance 140503). Lancet Respir. Med..

[21] Saji H., Okada M., Tsuboi M., Nakajima R., Suzuki K., Aokage K., Aoki T., Okami J., Yoshino I., Ito H. (2022). Segmentectomy versus lobectomy in small-sized peripheral non-small-cell lung cancer (JCOG0802/WJOG4607L): A multicentre, open-label, phase 3, randomised, controlled, non-inferiority trial. Lancet.

[22] Paul S., Altorki N.K., Sheng S., Lee P.C., Harpole D.H., Onaitis M.W., Stiles B.M., Port J.L., D’Amico T.A. (2010). Thoracoscopic lobectomy is associated with lower morbidity than open lobectomy: A propensity-matched analysis from the STS database. J. Thorac. Cardiovasc. Surg..

[23] Villamizar N.R., Darrabie M.D., Burfeind W.R., Petersen R.P., Onaitis M.W., Toloza E., Harpole D.H., D’Amico T.A. (2009). Thoracoscopic lobectomy is associated with lower morbidity compared with thoracotomy. J. Thorac. Cardiovasc. Surg..

[24] Farjah F., Flum D.R., Varghese T.K., Symons R.G., Wood D.E. (2009). Surgeon Specialty and Long-Term Survival After Pulmonary Resection for Lung Cancer. Ann. Thorac. Surg..

[25] Provencio M., Serna-Blasco R., Nadal E., Insa A., García-Campelo M.R., Casal Rubio J., Dómine M., Majem M., Rodríguez-Abreu D., Martínez-Martí A. (2022). Overall Survival and Biomarker Analysis of Neoadjuvant Nivolumab Plus Chemotherapy in Operable Stage IIIA Non–Small-Cell Lung Cancer (NADIM phase II trial). J. Clin. Oncol..

[26] Wu Y.L., Tsuboi M., He J., John T., Grohe C., Majem M., Goldman J.W., Laktionov K., Kim S.W., Kato T. (2020). Osimertinib in Resected EGFR-Mutated Non–Small-Cell Lung Cancer. N. Engl. J. Med..

[27] Kim S.S., Schumacher L., Cooke D.T., Servais E., Rice D., Sarkaria I., Yang S., Abbas A., Sanchetti M., Long J. (2025). The Society of Thoracic Surgeons Expert Consensus Statements on a Framework for a Standardized National Robotic Curriculum for Thoracic Surgery Trainees. Ann. Thorac. Surg..

[28] Moon Y., Choi S.Y., Moon M.H. (2020). The prognosis of stage I non-small cell lung cancer with visceral pleural invasion and whole pleural adhesion after video-assisted thoracoscopic lobectomy: A single center retrospective study. J. Thorac. Dis..

[29] Ma J., Li X., Zhao S., Wang J., Zhang W., Sun G. (2021). Robot-assisted thoracic surgery versus video-assisted thoracic surgery for lung lobectomy or segmentectomy in patients with non-small cell lung cancer: A meta-analysis. BMC Cancer.

